# Results of Tonsillectomy under Local Anesthesia

**Published:** 1913-07

**Authors:** 


### Abstracts and Selections.

### Results of Tonsillectomy Under Local Anesthesia.

Abstract of paper read at the Minneapolis meeting of the American Medical Association June 17-21, by Bryan DeForest Sheedy, M. D., of New York:

All of the one hundred cases reported upon by the reader of the paper were examined several months after operation, and no patient under fourteen years of age was operated upon under local anesthesia. There was no grouping of the patients examined as to whether throat conditions were the result of operation under local or general anesthesia. The enucleation of the tonsils had been performed by some one of the many methods in vogue for the last few years for the complete removal of the gland, and as the operations were performed in practically all the public institutions in New York city, many men of prominence in laryngology were the operators, so that the results could not be attributed to poor technique on the part of one man.

The writer arrived at the conclusion that tonsillectomy, so far as removing pathological tonsils is concerned, is a better operation than the old-time tonsillotomy, but pointed out that many of the throat defects following the operation of enucleation are due to clumsy and non-surgical technique.

The writer also pointed out the normal relation of the surrounding parts to the tonsil and put up a strong argument against the use of sharp instruments for the dissection of the tonsil from its bed, that being the cause of injury to the muscles with resulting deformities.

Of the one hundred cases examined months after operation more than 80 per cent of the patients had deformed throats. The 20

per cent of patients with what appeared to be normal throats following the operation were inconvenienced in no way at any time following the operation. Of the eighty patients thirty-four complained of speech defects for from one to three weeks after operation, sixteen complained of speech defects for more than three months after operation, while four had practically lost the singing voice. About 25 per cent of the patients stated that their throats felt better and that they could speak and sing better after operation than before. Inability to use certain words continued with 5 per cent of the patient for more than six months after operation.

The variety of deformities following enucleation were classified as follows:

1. The pillars on both sides had disappeared, with the soft palate tightened to such an extent that the opening at the nasopharynx was narrowed.

2. The pillars on both sides had grown together.

3. The anterior pillar had wholly disappeared, with a large amount of cicatricial tissue deposited on the posterior pillar.

In the four patients whose singing voice had been seriously affected the posterior pillar had disappeared through amalgamation with the anterior or with the lateral wall of the pharynx.

The reader emphasized the fact that he did not think the last word had been said in regard to tonsil enucleation and proposed as a remedy for preventing the unsatisfactory throat results an operation for removing the tonsil by what he called the "eversion method" and with charts and diagrams pointed out that the capsule of the tonsil is simply a bag, the bottom of which may be pulled through its mouth so that its inner surface becomes the outer, and that if the capsule, with its glandular tissue, is everted and a snare placed on, removing the tonsil with its capsule complete (there being no dissection and therefore no injury to the muscles surrounding), there would be no deformities.

The exceptions to the rule presented, viz., that the tonsil will evert on traction, were:

1. Those cases in which the capsule was bound down to the surrounding tissues by previous attacks of inflammation.

2. Those cases where the capsule was very much contracted and contained cicatricial tissue only.

3. Those cases of hypertrophied tonsils which had everted themselves and the tonsil was found everted when the patient applied for treatment.

The points advanced in favor of the procedure were:

1. Simplicity of the operation.

2. Practically no hemorrhage.
3. Little or no deformity following the procedure.
4. Only three instruments necessary for the operation, viz., tonsil tenaculum, blunt-pointed tonsil knife, Tyding snare.

